# Coinfection with a virus constrains within‐host infection load but increases transmission potential of a highly virulent fungal plant pathogen

**DOI:** 10.1002/ece3.8673

**Published:** 2022-03-08

**Authors:** Hanna Susi, Suvi Sallinen, Anna‐Liisa Laine

**Affiliations:** ^1^ 27217 Research Centre for Ecological Change, Organismal and Evolutionary Biology Research Programme University of Helsinki Helsinki Finland; ^2^ 27217 Department of Evolutionary Biology and Environmental Studies University of Zurich Zurich Switzerland

**Keywords:** cross‐kingdom interactions, epidemiology, evolution, host‐pathogen interactions, life‐history traits, parasites

## Abstract

The trade‐off between within‐host infection rate and transmission to new hosts is predicted to constrain pathogen evolution, and to maintain polymorphism in pathogen populations. Pathogen life‐history stages and their correlations that underpin infection development may change under coinfection with other parasites as they compete for the same limited host resources. Cross‐kingdom interactions are common among pathogens in both natural and cultivated systems, yet their impacts on disease ecology and evolution are rarely studied. The host plant *Plantago lanceolata* is naturally infected by both *Phomopsis subordinaria*, a seed killing fungus, as well as *Plantago lanceolata latent virus* (PlLV) in the Åland Islands, SW Finland. We performed an inoculation assay to test whether coinfection with PlLV affects performance of two *P*. *subordinaria* strains, and the correlation between within‐host infection rate and transmission potential. The strains differed in the measured life‐history traits and their correlations. Moreover, we found that under virus coinfection, within‐host infection rate of *P*. *subordinaria* was smaller but transmission potential was higher compared to strains under single infection. The negative correlation between within‐host infection rate and transmission potential detected under single infection became positive under coinfection with PlLV. To understand whether within‐host and between‐host dynamics are correlated in wild populations, we surveyed 260 natural populations of *P*. *lanceolata* for *P*. *subordinaria* infection occurrence. When infections were found, we estimated between‐hosts dynamics by determining pathogen population size as the proportion of infected individuals, and within‐host dynamics by counting the proportion of infected flower stalks in 10 infected plants. In wild populations, the proportion of infected flower stalks was positively associated with pathogen population size. Jointly, our results suggest that the trade‐off between within‐host infection load and transmission may be strain specific, and that the pathogen life‐history that underpin epidemics may change depending on the diversity of infection, generating variation in disease dynamics.

## Graphical abstract

We found that under virus coinfection, within‐host infection load of *Phomopsis subordinaria* was lower but transmission potential was higher compared to strains under single infection. The negative correlation between within‐host infection load and transmission potential detected under single infection became positive under coinfection with virus. In wild populations, within‐host infection load was positively associated with within‐population disease prevalence.

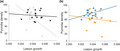

## 1 INTRODUCTION

To grow, multiply, and transmit, pathogens obtain resources from their host, and theoretically the resulting within‐host infection rate is expected to be proportional to the gained resources. However, too high within‐host infection rate may come at the cost of increased virulence, that is measurable damage caused to the host (Surico, 2013), thereby incurring a cost for the pathogen as decreased transmission to other hosts (Blanquart et al., 2016). Thus, evolutionary theory and experimental studies (de Roode et al., 2008) have established that selection should favor intermediate levels of within‐host infection rates. This trade‐off between within‐host infection rate and transmission has been proposed to maintain polymorphism in pathogen populations, and to prevent the rise of highly virulent pathogens (Frank, 1992). Trade‐offs have been sought as an evolutionary solution to limit disease epidemics and the emergence of pathogen strains with extremely high within‐host infection rate (Zhan et al., 2015). However, insight on how pathogen within‐host infection rate links to transmission during epidemics where pathogens may encounter variation in both biotic and abiotic conditions (Blanquart et al., 2016; Dutta et al., 2021; Susi & Laine, 2013) has remained limited (Acevedo et al., 2019). In the wild, the limited evidence for trade‐offs may be explained by spatial (Osnas et al., 2015) and host‐mediated processes (Kubinak et al., 2012). The trade‐offs restraining within‐host infection rate may also occur between other traits, that is, adaptation to abiotic conditions (Mboup et al., 2012) or be context‐dependent and become evident in stressful environments (Susi & Laine, 2013).

The drivers of disease evolution and epidemics are rarely limited to the interplay of one host and one pathogen, as in the wild most infections occur as coinfections whereby multiple pathogens are simultaneously infecting the same host (Telfer et al., 2010; Tollenaere et al., 2016). Coinfection may fundamentally change pathogen's host exploitation strategy in order to outcompete other pathogens sharing the same limited resource (Alizon & van Baalen, 2008; Alizon et al., 2009; de Roode et al., 2005). Thus, it has been suggested that coinfection is an important driver of disease evolution (Alizon & van Baalen, 2008; Alizon et al., 2009). Experimental approaches have measured increased within‐host infection rate (Bell et al., 2006) and transmission (Susi et al., 2015; Susi et al., 2015) under coinfection but there are also exceptions to this trend (Orton & Brown, 2016). Overall, it is well established that the pathogen within‐host infection rate may change under coinfection, but studies explicitly testing trade‐offs between within‐host infection rate and transmission under coinfection are rare, and evidence remains mixed (Sacristan & Garcia‐Arenal, 2008; Suffert et al., 2016). Furthermore, coinfection experiments have often been conducted using strains of the same pathogen species, although interspecific interactions among pathogen species are likely to play an important role, as individual hosts often support diverse pathogen assemblages (Dallas et al., 2019; Susi et al., 2019; Telfer et al., 2010).

While intraspecific coinfection is a pre‐requisite for outcrossing for many pathogens (Suffert et al., 2016), theory predicts the intensity of competition to increase as relatedness decreases (Alizon et al., 2013). Across plants (Tollenaere et al., 2016, 2017), animals (Telfer et al., 2010) and humans (Chen et al., 2020; Lawn et al., 2006) inter‐kingdom coinfections are common, and they are often suggested to have serious consequences for disease epidemics and severity. In particular, it is becoming increasingly clear that viruses are ubiquitous in nature (Bernardo et al., 2017; Munson‐McGee et al., 2018), although their true diversity and prevalence in natural populations has been under‐estimated for a long time (Roossinck et al., 2015; Wren et al., 2006). The ecological roles of viruses are still poorly understood (Alexander et al., 2014; Roossinck, 2010), but they have the potential to interact with other pathogen species via competition for shared host resources, and via shared effects on host immunity (Huang et al., 2019; Uehling et al., 2017). Thus, it is vital to test how coinfection with pathogens from distant taxa may influence within‐host infection rate and transmission, and their potential trade‐offs.

*Phomopsis subordinaria* is a castrating pathogen that infects its hosts through seed stalks. Potential trade‐offs between within‐host infection rate and transmission would translate in field epidemics as reduced transmission in the populations with high within‐host infection rates. Here, we investigate the association between within‐host dynamics and between‐host dynamics of *P*. *subordinaria* by surveying 260 host plant (*Plantago lanceolata*) populations in the Åland Islands, south‐west Finland. We combined data from the field measured disease with laboratory trials. In the laboratory, we challenged *P*. *subordinaria* strains with *Plantago lanceolata latent virus* (PlLV) to understand how cross‐kingdom interactions affect within‐host infection rate and transmission potential as well as their potential trade‐off. Specifically, we ask (1) How common is *P*. *subordinaria* in the Åland Islands, and is there natural variation in the within‐host infection dynamics in natural *P*. *lanceolata* populations, and (2) Is there an association between within‐host and between‐host dynamics in *P*. *subordinaria*? In a laboratory experiment, we tested: (3) Is there a trade‐off between within‐host infection rate and transmission potential in *P*. *subordinaria*? We hypothesize that high within‐host infection rate is costly in transmission potential. (4) Does coinfection with PlLV alter *P*. *subordinaria* within‐host infection rate and transmission potential? We hypothesize that coinfection increases within host infection rate and transmission potential.

## 2 MATERIALS AND METHODS

### 2.1 Host plant and the pathogens

*Plantago lanceolata* is a perennial wind pollinated rosette‐forming herb (Sagar & Harper, 1964) with a worldwide distribution. In the Åland Islands, it occurs as a network consisting of ca. 4000 meadows in a highly fragmented landscape (Ojanen et al., 2013). Annually in September, the 4000 *P*. *lanceolata* populations' size, area, and location are monitored and the presence of *Podosphaera plantaginis* fungus and *Melitaea cinxia* butterfly are surveyed. (Jousimo et al., 2014; Ojanen et al., 2013). Due to the fragmented landscape in the Åland Islands, *P*. *lanceolata* populations are discrete patches surrounded by unsuitable matrix consisting of rocky outcrops, dense forests, waterbodies, or agricultural fields where the plant cannot grow (Ojanen et al., 2013). The patches are separated by minimum distance of 20 meters of non‐suitable habitat or 50 meters of suitable area without *P*. *lanceolata* (Ojanen et al., 2013). Each patch is GPS delineated and their average size is 0.5 hectares (Ojanen et al., 2013). In the Åland Islands *P*. *lanceolata* has been discovered to host two fungal pathogens, *P*. *plantaginis* and *Phomopsis subordinaria* (Jousimo et al., 2014; Laine, 2003) and five viruses (Susi et al., 2019) thus far. *Phomopsis subordinaria* (telemorph *Diaporthe adunca* (Rob.) Niessl.) is a specialist fungal pathogen of *P*. *lanceolata* (Laine, 2003) transmitted by weevil *Trichosirocalus troglodytes* (Nieminen & Vikberg, 2015; de Nooji & van der Aa, 1987). The pathogen infects its host plant through a wound under the inflorescence of the plant causing the developing seeds to dry out (de Nooji & van der Aa, 1987). The pathogen kills the plant cells and feeds on dead tissue, subsequently causing death of the whole plant. It produces pycnidia fruiting bodies in which its spores are formed. In our laboratory trial, we use pycnidia density as a measure of transmission potential of the pathogen, more abundant pycnidia formation can be expected to also increase spore load carried by the weevil vector. *Plantago lanceolata latent virus* (PlLV) is a DNA virus belonging to Capulaviruses in Geminiviridae (Susi et al., 2017). The virus has been recently characterized (Susi et al., 2017, 2019) and it is relatively common in populations of *P*. *lanceolata* in the Åland Islands (33% of populations infected) (Susi & Laine, 2021). Mode of transmission and potential host range of PlLV are currently unknown.

### 2.2 *Phomopsis* *subordinaria* field survey

To characterize the distribution and drivers of *P*. *subordinaria*, and to measure the relationship between within‐host and between‐host dynamics, we surveyed 260 *P*. *lanceolata* populations in early September 2018 for infection by *P*. *subordinaria*. These populations were selected to represent different areas in the Åland Islands. The field identification of *P*. *subordinaria* was confirmed by microscopy of field collected samples consisting of an infected flower stalk. To understand possible correlations between within‐host and between‐host dynamics, we examined two measures in the field populations. First, we used proportion of flower stalks infected within a plant as a measure of within‐host dynamics because it directly quantifies the harm caused to a susceptible host as the death of the seeds. *Phomopsis subordinaria* infection spreads from an infected flower stalk through rosette and eventually kills its host plant within 7–10 weeks (de Nooij & Damme, 1988a). Highly virulent infection will thus prevent the host from producing any seeds whereas lower virulence of the pathogen would allow the plant to produce greater proportion of healthy stalks and seed. Secondly, we measured between‐host dynamics by counting the infected plants and estimating the pathogen population size in each infected *P*. *lanceolata* population as the proportion of infected plants (1 = 0.01–0.1 infected plants; 2 = 0.11–0.25 infected plants; 3 = 0.26–0.5 infected plants; and 4 = 0.51–1 infected plants). Proportion of infected plants in a population reflects the realized transmission for pathogens that rely on horizontal transmission. We used a categorical scale in estimating the pathogen population size because it is the most feasible way of estimating disease in the field, and especially suitable in the analysis of low disease prevalence (Chiang & Bock, 2021). Host population size is expected to increase infection risk of populations (Parratt et al., 2016) and thus, we estimated the host population size as coverage of the host plant in square meters. Host population connectivity was measured in order to understand how distances between host populations may impact pathogen prevalence (Jousimo et al., 2014). Host population connectivity was calculated as

$$S_{i}^{L}=\sum \exp\left(‐\alpha d_{\mathrm{ij}}\right)\surd A_{j},$$

where *d_ij_* is the Euclidian distance between patches *j* and *i*, and *α* is the parameter of the negative exponential dispersal kernel, which was set to 1 km^−1^ (see Jousimo et al. (2014) for more details). *A_j_*_,_ is the square root transformation of area (m^2^) of habitat patch *j*.

The Åland archipelago is highly fragmented and consists of the main island and smaller islands that jointly form 16 regional districts of similar size. To capture possible spatial variation among the regions within the islands, we used regional district as a spatial unit. In our sampling area, there were nine regional districts (Eckerö (12), Finström (41), Hammarland (38), Jomala (10) Lemland (37), Lumparland (49), Maarianhamina (11), Saltvik (19), and Sund (46), with the number of visited populations within each regional district given in parenthesis).

### 2.3 Inoculation experiment

To investigate potential trade‐offs between within‐host infection rate and transmission potential, and whether coinfection with PlLV affects *P*. *subordinaria* infection or trade‐offs, we performed a laboratory trial using two *P*. *subordinaria* strains (P29 and P43 originating from populations 1720 and 861, respectively), one PlLV strain and one *P*. *lanceolata* genotype (511–14) originating from the Åland Islands. Altogether 32 plants were used in the experiment. To compare *P*. *subordinaria* performance and trade‐offs alone and in coinfection with PlLV, we inoculated half of the plants with a PlLV strain originating from population 3301 and maintained in *P*. *lanceolata*, whereas half of the plants received mock inoculation with phosphate buffer. Sap from PCR confirmed (Susi et al., 2017) PlLV‐infected plants mixed with phosphate buffer was used for virus inoculation. Using a syringe, one leaf per plant was inoculated with 100 µl virus sap or phosphate buffer. After 7 days from PlLV inoculation, the plants received *P*. *subordinaria* inoculation. To understand pathogen strain effect on trade‐offs with PlLV, half of the plants received an inoculation with strain P29, and half received an inoculation with strain P43. The fungal strains were collected from the field, purified on sequential inoculations on oat agar plates (de Nooji & van der Aa, 1987) and maintained on live *P*. *lanceolata* plants. Up to four flower stalks per plant were inoculated by wounding the stalk just below the inflorescence with a scalpel and immediately pipetting 10^6^ conidia/µl suspension to the wound. In the experiment, each treatment (PlLV coinfection yes/no with *P*. *subordinaria* strain P29 or P43) was replicated eight times. The plants were kept inside a growth chamber in 8:16 dark: light cycle at +20°C. To prevent possible shelf effects, the locations of plants inside the chamber were moved daily. To quantify within‐host infection rate, we measured the size of the *P*. *subordinaria* lesions because lesion size correlates negatively with seed production (de Nooij & Van der Aa, 1987). We measured the lesion and the length of each flower stalk in centimeters once a week starting 7 days post‐inoculation until 4 weeks post‐inoculation (de Nooij & Damme, 1988b). The size of the lesion was then calculated by dividing the lesion size with the length of the flowers stalk. Lesion growth measurements were then used for calculation of relative area under disease progress stairs (AUDPS; (Simko & Piepho, 2012)). To measure transmission potential, we observed pycnidia density. Pycnidia are the fruiting bodies of the fungus that contain the conidial spores that spread the fungus vectored by the weevil *T*. *troglodytes* (de Nooji & van der Aa, 1987). Pycnidia density measures the capacity of the fungus to produce transmission propagules to be transmitted from plant to plant by the weevil vector, and hence, we consider it as a reasonable proxy for transmission potential. At the end of the experiment, 4 weeks past *P*. *subordinaria* inoculation, we counted the pycnidia density within a square centimeter on the flower stalk that produced the first pycnidia from each plant using a microscope.

### 2.4 Statistical analyses

To analyze drivers of *P*. *subordinaria* epidemics in *P*. *lanceolata* populations in the Åland Islands, we ran Generalized linear models in SAS Proc Glimmix software (SAS Institute Inc.). We first fit a model with infection occurrence in the host population (0 = no infection, 1 = infection) as a binary response variable, and host population size and connectivity as covariates, and regional district as categorical explanatory variable. Including host population connectivity allows controlling for spatial variation in putative gene flow among populations (Hanski, 1999), and including regional district allows controlling for possible regional variation in these data. To understand factors explaining *P*. *subordinaria* population size within host populations (estimate of between‐host dynamics), we fit a second model with the within population prevalence, that is, proportion of infected plants within host populations as a class response variable (1 = 0.01–0.1 infected plants; 2 = 0.11–0.25 infected plants; 3 = 0.26–0.5 infected plants; and 4 = 0.51–1 infected plants), host population size and connectivity as covariates, and regional district as a categorical explanatory variable. We fit a third model to understand how the proportion of infected flower stalks (estimate of within‐host dynamics) and pathogen population size (estimate of between‐host dynamics) are linked in natural populations. We used the proportion of infected flower stalks as response variable and included the within population prevalence as average proportion of infected stalks in a population as explanatory variable, and, host population connectivity, host population size, and regional district as covariates. A Gamma distribution of errors was assumed. We did not include interactions between covariates in the models as they were not biologically meaningful.

We then analyzed the results from the laboratory trial measuring the performance of two *P*. *subordinaria* strains alone and in coinfection with PlLV using generalized linear mixed models implemented in SAS Proc Glimmix software (SAS Institute Inc.). To test whether PlLV coinfection influences *P*. *subordinaria* lesion growth (within‐host infection rate), we used lesion growth as a response variable with a Gaussian distribution of errors. In this model, we used *P*. *subordinaria* strain and virus inoculation treatment (1 = PlLV inoculation and 0 = mock inoculation) as categorical explanatory variables. Flower stalk (*n* = 90) was nested under plant individual (*n* = 32) and used as random factor. To understand how virus coinfection and *P*. *subordinaria* strain identity affect pycnidia density, we analyzed the subset of stalks from which the pycnidia were counted (*n* = 32) using a generalized linear model. Number of pycnidia was used as a continuous response variable and PlLV coinfection and *P*. *subordinaria* strain as categorical explanatory variables. Poisson distribution of error was assumed. Odds ratios were calculated for virus coinfection and pathogen strain. In both models, interaction between virus coinfection and pathogen strain was tested and only statistically significant interactions were kept in the model.

To test for possible trade‐offs between lesion growth and pycnidia density, we ran a model using the stalks (*n* = 32) from which pycnidia were counted in a generalized linear model in SAS Proc Glimmix software (SAS Institute Inc.). We included lesion growth as a covariate, and virus inoculation treatment (1 = PlLV inoculation and 0 = mock inoculation) and *P*. *subordinaria* strain as categorical explanatory variables. The response variable in the model was the pycnidia density measured as the number of pycnidia number counted in 1 cm^2^ area on the lesions. A Poisson distribution of errors was assumed. Odds ratios were calculated for virus coinfection and pathogen strain. Interactions between virus coinfection and pathogen strain and other explanatory variables were tested, and only statistically significant interactions were kept in the model.

## 3 RESULTS

### 3.1 *Phomopsis subordinaria* field survey

**FIGURE 1:**
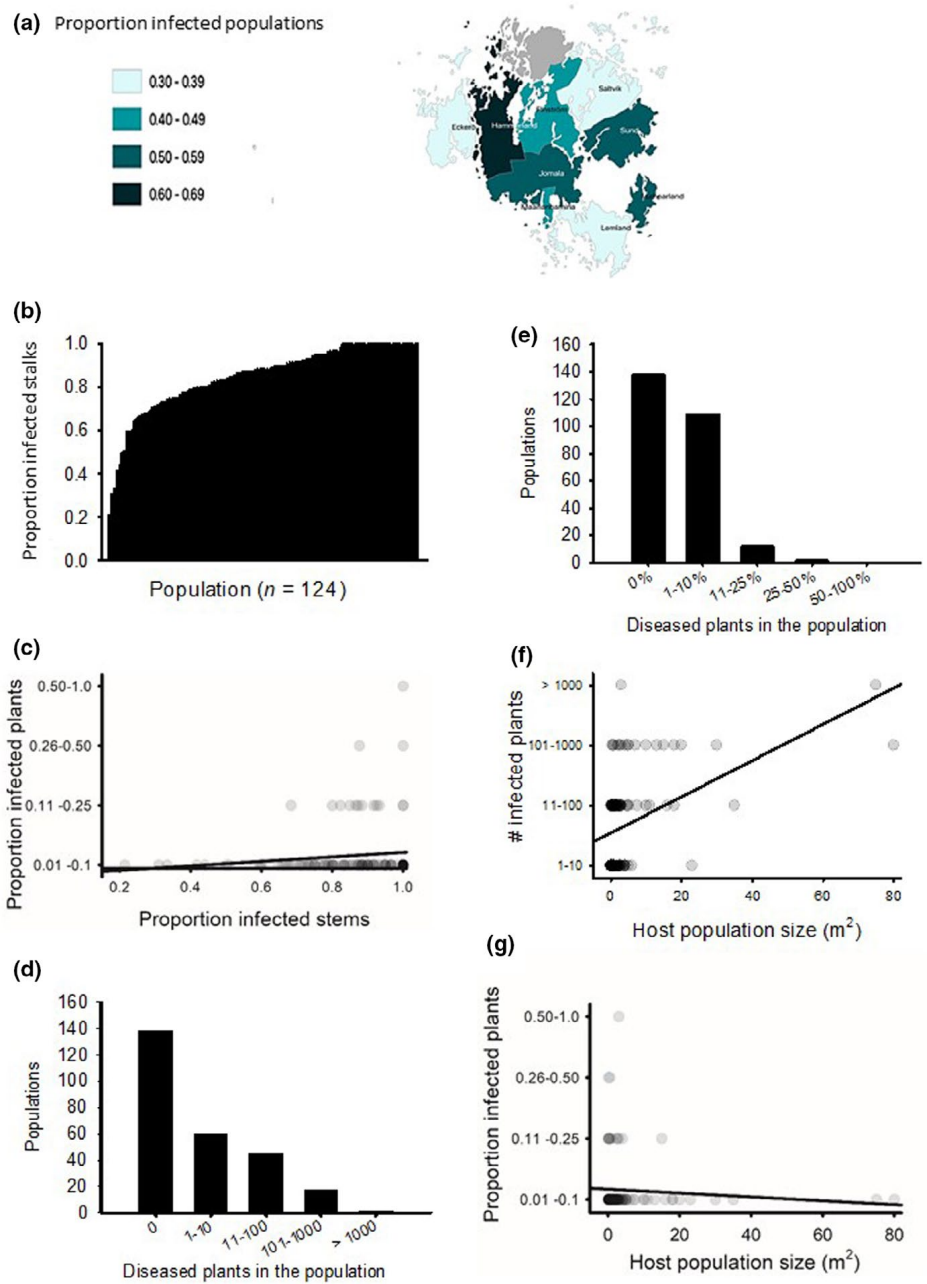
Variation in *Phomopsis subordinaria* infection prevalence in 260 *Plantago lanceolata* populations in the Åland Islands. (a) The proportion of infected populations in the nine regional districts surveyed. The effect of host population (b) size and (c) connectivity on infection prevalence. (d) The mean within‐host infection rate in infected populations measured as proportion of infected stalks within each infected plant. The pathogen within‐population prevalence (e) as the number of infected plants in the populations, and (f) as the proportion of infected plants in the populations. (g) The relationship between pathogen within‐population prevalence in the population of infection and within‐host infection rate

We found that *P*. *subordinaria* was widely spread in *P*. *lanceolata* populations in the Åland Islands as in nearly half of the (124) of the sampled 260 populations; one or more infected plants were found (Figure 1a). Our first model testing infection occurrence across the populations showed that large populations were more commonly infected than small, and host population connectivity was positively associated with *P*. *subordinaria* infection (Table 1, Figure 1b,c). We also found regional spatial variation in infection prevalence that was the highest in Hammarland, where 25 of the 37 surveyed host populations were infected, and lowest in Saltvik where six of the 19 surveyed host populations were infected (Table 1, Figure 1a).

**TABLE 1:** Results from Generalized Linear Models on *Phomopsis subordinaria* field survey across 260 natural *Plantago lanceolata* populations across the Åland Islands, and from a laboratory inoculation experiment testing the impact of virus coinfection and strain identity on performance of the pathogen, as well as life‐history trade‐offs. Statistically significant values (*p* < .05) are shown in bold

*Phomopsis subordinaria* epidemics	Occurrence in populations		
	*df*	*F*	*p*
Connectivity	**1, 247**	**9.81**	.**0019**
Regional district	**8, 247**	**2.71**	.**0071**
Host population size	**1, 247**	**8.95**	.**0031**

*Phomopsis subordinaria* epidemics	Pathogen population size			The proportion of infected stalks		
	*df*	*F*	*p*	*df*	*F*	*p*
Connectivity	1, 109	1.06	.3057	1, 109	0.15	.701
Regional district	**8, 109**	**5.4**	**<.0001**	8, 109	0.23	.985
Host population size	1, 109	0.61	.4353	1, 109	0	.952
The proportion of infected stalks	1, 109	**4.03**	.**0471**			
Within population prevalence				1, 109	0.49	.485

Laboratory performance	Lesion growth			Odds ratios		Pycnidia density			Odds ratios	
	*df*	*F*	*p*	*Estimate*	*St. error*	*df*	*F*	*p*	*Estimate*	*St. error*
Virus coinfection	**1, 28**	**8.7**	.**0064**	**0.000675**	**0.00023**	**1, 29**	**8.41**	.**007**	**−0.2289**	**0.04282**
Strain	**1, 28**	**11.04**	.**0025**	**−0.00075**	**0.00023**	1, 29	0.16	.6962	0.03103	0.04254

Laboratory measured trade‐offs	Pycnidia density			Odds ratios	
	*df*	*F*	*p*	*Estimate*	*St. error*
Lesion growth	1, 26	2.67	.1145		
Strain identity	**1, 26**	**9.98**	.**004**	**−0.04704**	**0.04875**
Virus coinfection	1, 26	1.07	.3099	0.198	0.2018
Lesion growth × strain	**1, 26**	**5.11**	.**0323**		
Lesion growth × virus coinfection	**1, 26**	**11.73**	.**002**		

Within infected host populations, *P*. *subordinaria* population size was typically low, but the proportion of infected flower stalks was high (Figure 1d). In nearly half of the populations, we found one to 10 infected plants (Figure 1e). In one‐third of the infected populations, 11–100 infected plants were found, and in only small fraction of populations we found more than 1000 infected individuals (Figure 1e). In most infected host populations less than 10% of all plants were infected, and in only small fraction of the populations we observed more than 25% of infected plants (Figure 1f). At the plant level, we found that *P*. *subordinaria* infections were highly virulent. On average, 85% of stalks were infected within infected plants (Figure 1d) while the mean number of flowers per plant was 2.0 and median was 2 (min 1; max 22).

Our second model testing which factors affect *P*. *subordinaria* population size, regional districts differed in the pathogen population size, and the proportion of infected stalks had small but significant positive effect on pathogen population size (Table 1; Figure 1g). Neither host population connectivity nor size influenced *P*. *subordinaria* population size (Table 1). None of the tested variables—regional district, host population connectivity and size, *P*. *subordinaria* population size, or within‐population prevalence—had a significant effect on proportion of infected flower stalks (within‐host infection rate) tested in our third model (Table 1).

### 3.2 Coinfection with virus alters *Phomopsis subordinaria* performance

**FIGURE 2:**
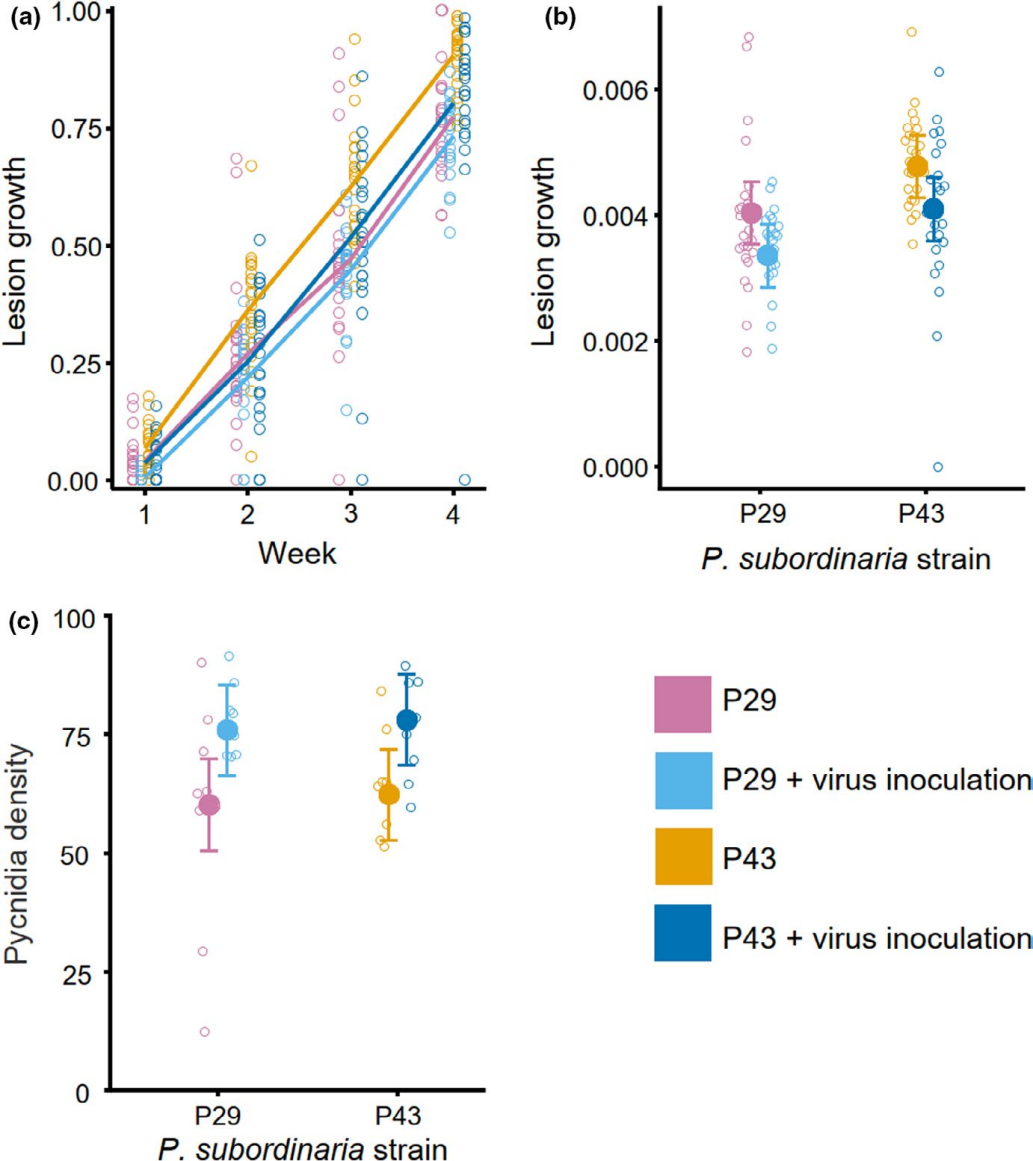
The impact of *Plantago lanceolata latent virus* (PlLV) infection on two strains of *Phomopsis subordinaria* performance on *Plantago lanceolata* in a laboratory experiment. (a) Mean proportion of each inoculated stalk (*n* = 90) with necrotic symptom (empty circle) with and without PlLV coinfection over the 4 weeks of data recording. Means of each time point for each treatment are visualized with a line. (b) Variation in lesion growth among *P*. *subordinaria* strains with and without coinfection with PlLV. Empty circles represent each inoculated flower stalk (*n* = 90) and mean + standard error from a linear model are presented for each treatment. (c) Variation in pycnidia density among *P*. *subordinaria* strains with and without coinfection with PlLV. Empty circles represent each inoculated flower stalk (*n* = 32) and mean + standard error from a linear model are presented for each treatment

We investigated the effect of PlLV coinfection on *P*. *subordinaria* within‐host infection rate (measured as lesion growth), and transmission potential (measured as pycnidia density) in an inoculation experiment with two *P*. *subordinaria* strains (P29 and P43). The two strains differed significantly in their lesion growth with strain P43 outperforming strain P29 (Table 1; Figure 2a,b). For both strains, lesion growth was lower under coinfection with PlLV than when *P*. *subordinaria* infected the host alone (Table 1; Figure 2a,b). Pycnidia density was significantly higher under coinfection than under single infection, while *P*. *subordinaria* strains did not differ in their pycnidia densities (Table 1; Figure 2c).

### 3.3 Trade‐offs between life‐history traits

**FIGURE 3:**
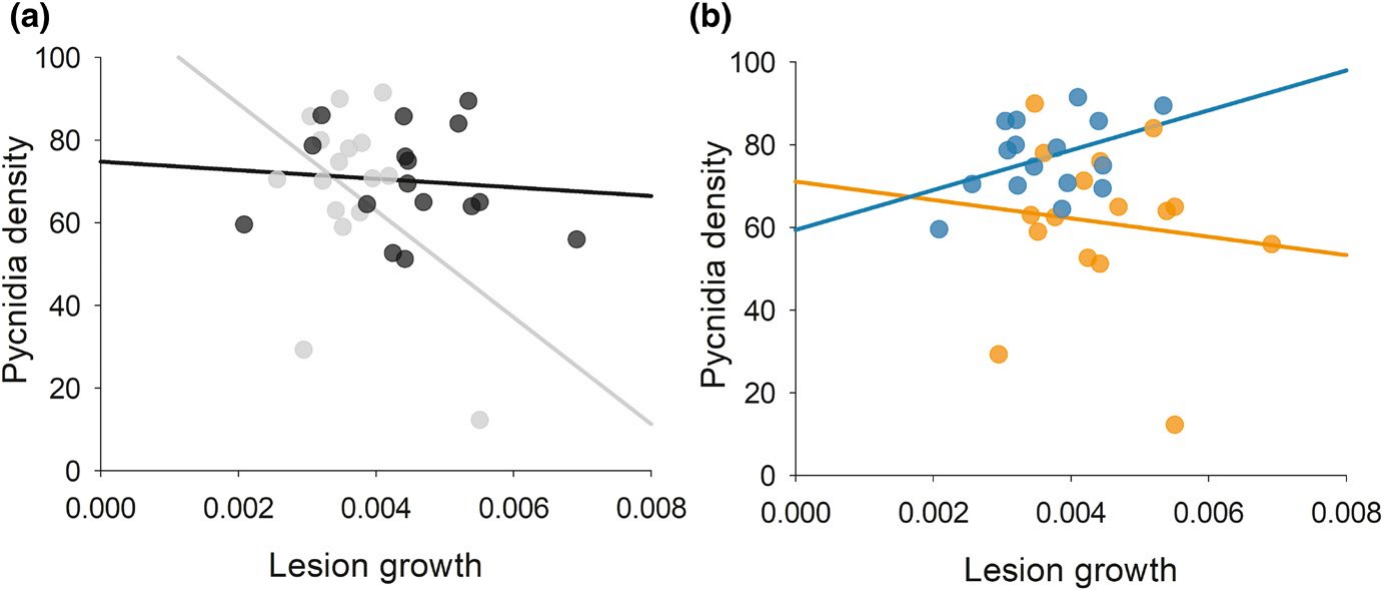
The impact of strain identity and *Plantago lanceolata latent virus* (PlLV) coinfection on life‐history trade‐offs in *Phomopsis subordinaria* on *Plantago lanceolata* in a laboratory inoculation trial. The impact of strain identity (grey = strain P29; black = strain 43) relationship between (a) pycnidia density and lesion growth. (b) The impact of PlLV coinfection (blue) versus mock inoculation (orange) on the relationship between pycnidia density and lesion growth. The lines show univariate regression

Finally, we tested whether there are trade‐offs between lesion growth and pycnidia density, and whether *P*. *subordinaria* strain or PlLV coinfection influence trade‐offs. In this model, pathogen strain had significant impact on pycnidia density, but PlLV coinfection did not (Table1). We found that while pycnidia density was not directly correlated with lesion growth, the relationship between pycnidia density and lesion growth was mediated by strain identity (significant interaction lesion growth ×strain; Table 1; Figure 3a) and by coinfection with PlLV (significant interaction lesion growth ×PlLV coinfection; Table 1; Figure 3b). There was a negative correlation between lesion growth and pycnidia density in strain P29 suggesting a trade‐off, whereas in strain P43 there was no evidence of a trade‐off (Figure 3a). Similarly, in mock inoculated plants trade‐offs between pycnidia density and lesion growth was observed, but not in PlLV infected plants (Figure 3b).

## 4 DISCUSSION

Although the trade‐off between within‐host infection rate and transmission is a central tenet of pathogen evolution (Alizon et al., 2009), remarkably little is understood of this association in natural populations, and under coinfection scenarios that are prevalent across pathosystems (Alizon et al., 2013; Tollenaere et al., 2016). Here, we studied within‐host infection rate and between‐host transmission potential experimentally to understand how sensitive their association is to pathogen strain identity and coinfection with a virus. We conduct a survey of infection across 260 wild host populations to test whether our experimental results are reflected in epidemiological patterns in the wild.

*Phomopsis subordinaria* was detected in nearly half of the surveyed natural host populations. The other fungal pathogen studied in this same host population network—the powdery mildew fungus *P*. *plantaginis*—infects annually 2–20% of *P*. *lanceolata* populations (Jousimo et al., 2014), and hence, by comparison *P*. *subordinaria* is relatively common. In few other wild plant pathosystems, similar disease incidence rates have been observed (e.g., *Triphragmium ulmariae* rust infected 29–69% *Filipendula ulmaria* host populations (Zhan et al., 2018) and *Uromyces valerianae* rust infected 43–73% of *Valeriana salina* populations (Ericson et al., 1999)). In our study, spatial structure was the main determinant for pathogen incidence as both host population connectivity and regional district explained variation in *P*. *subordinaria* distribution. Positive correlation between host connectivity and infection occurrence suggests that host population connectivity increases between population transmission, as predicted by metapopulation theory (Hanski, 1999). Large populations were more likely to be infected than small populations, which is also in line with theoretical predictions (Hanski, 1999). Unlike in *P*. *plantaginis* (Jousimo et al., 2014), host population size did not have an effect on pathogen population size. While airborne pathogens such as *P*. *plantaginis* are expected to be sensitive to host population size through density‐dependent transmission, *P*. *subordinaria* is vector‐transmitted and hence, it is expected to be less responsive to variation in host population size (Thrall et al., 1995). This result highlights the importance of the mode of transmission for epidemics.

Our field survey revealed that in infected host plants the majority inflorescences were infected (on average 85% of inflorescences), while in most infected host populations less than 10% of all plants were infected. We did not find evidence of a trade‐off between within‐host infection rate and transmission limiting the spread of *P*. *subordinaria* within its host populations. Instead, we found a positive correlation between within‐host rate and between‐host dynamics. The observed low within‐host populations prevalence of *P*. *subordinaria* could also result from a range of other factors unrelated to life‐history correlations, including variation in host plant susceptibility or pathogen infectivity (de Nooij & Damme, 1988a), constrained vector transmission (de Nooij, 1988; Pleydell et al., 2018), and interactions with other pathogens than PlLV (Susi, Barres, et al., 2015; Susi, Vale, et al., 2015).

Cross‐kingdom coinfections are common (Chen et al., 2020; Lawn et al., 2006) (Telfer et al., 2010; Tollenaere et al., 2016, 2017), and they are often suggested to have serious consequences in disease epidemics, disease severity, and pathogen evolution. Cross‐kingdom coinfections may drive virulence evolution through virulence transmission trade‐offs (Alizon et al., 2009). The trade‐offs observed under single host–single pathogen scenarios may change under coinfection, as host exploitation rates are expected to change under coinfections (Alizon et al., 2009). *Plantago lanceolata* is a host for a number of pathogens in the Åland Islands, and coinfections are frequently observed (Susi, Barres, et al., 2015; Susi et al., 2019). To understand how coinfections and genotypic variation may shape life‐history correlations and disease dynamics of *P*. *subordinaria*, we studied disease development under coinfection with a recently characterized virus, PlLV, using two *P*. *subordinaria* strains. We found that coinfection with virus had a profound impact on within‐host infection rate and transmission potential of *P*. *subordinaria* that can further impact evolution and epidemiology of the pathogen. Coinfection alleviated the harm caused to the host and increased transmission potential. Under natural epidemics such trade‐offs could translate into low within‐host infection rate and increased among host transmission. Furthermore, in the wild the pathogens may attack the plant multiple times or in reverse arrival order compared to our experiment. Investigating the consequences of different arrival scenarios is an interesting avenue of future research.

The two strains differed significantly in their within‐host infection rate, with strain P43 outperforming strain P29. Significant variation among pathogen strains in their life‐history traits is commonly observed in natural pathogen populations (Tack et al., 2012). For both strains, within‐host infection rate was lower under coinfection with PlLV than when *P*. *subordinaria* infected the host alone, while transmission potential was significantly higher under coinfection than under single infection. The relationship between within‐host infection rate and transmission potential was mediated by both strain identity and coinfection. We observed a negative correlation between the measured life‐history stages in strain P29 suggesting a trade‐off, whereas in strain P43 there was no evidence of a trade‐off. This result is in line with previous research that found life‐history correlations to be depending on the pathogen genotype (Bruns et al., 2014; Clement et al., 2012). The negative relationship between lesion growth and pycnidia density became positive under coinfection. This is in line with an earlier study testing coinfection with two strains of powdery mildew fungus *P*. *plantaginis* where the strains had higher performance and positive life‐history trait correlations when challenged with a competing strain (Laine & Mäkinen, 2018). Contrary to other studies on coinfection where response to coinfection has been found pathogen strain specific, here the response of the *P*. *subordinaria* strains was similar. In bacteria–fluke coinfections on salmons (Louhi et al., 2015), genotype specific responses on coinfection were observed. Similarly, virus strain combinations resulted in different within‐host growth rates of bacterial pathogen on rice (Tollenaere et al., 2017).

This study increases our understanding on the factors generating diversity in epidemics in natural populations. Furthermore, this is one of the very first reports addressing the knowledge gap on how pathogen life‐history traits correlate in realized epidemics. By showing that pathogen coinfection and strain identity may alter life history correlations this study contributes to better understanding of disease evolution and epidemiology.

## CONFLICT OF INTEREST

The authors have no conflicts of interest to declare.

## AUTHOR CONTRIBUTIONS

**Hanna Susi:** Conceptualization (lead); Data curation (lead); Formal analysis (equal); Investigation (lead); Supervision (lead); Visualization (equal); Writing – original draft (lead). **Suvi Sallinen:** Data curation (supporting); Formal analysis (equal); Investigation (supporting); Methodology (supporting); Visualization (equal). **Anna‐Liisa Laine:** Funding acquisition (lead); Supervision (supporting).

## Data Availability

The data underlying the results presented in the study are available from Dryad (https://doi.org/10.5061/dryad.4mw6m90c9).
